# Regional disparities in SARS-CoV-2 infections by labour market indicators: a spatial panel analysis using nationwide German data on notified infections

**DOI:** 10.1186/s12879-022-07643-5

**Published:** 2022-07-30

**Authors:** Morten Wahrendorf, Marvin Reuter, Jens Hoebel, Benjamin Wachtler, Annika Höhmann, Nico Dragano

**Affiliations:** 1 Institute of Medical Sociology, Centre for Health and Society, Medical Faculty, Heinrich-Heine-University of Düsseldorf, Moorenstrasse 5, 40225 Düsseldorf, Germany; 2 Unit of Social Determinants of Health, Department of Epidemiology and Health Monitoring, Robert Koch Institute, Berlin, Germany

**Keywords:** Spatial analyses, Regional differences, Labour market, SARS-CoV-2

## Abstract

**Background:**

Regional labour markets and their properties are named as potential reasons for regional variations in levels of SARS-CoV-2 infections rates, but empirical evidence is missing.

**Methods:**

Using nationwide data on notified laboratory-confirmed SARS-CoV-2 infections, we calculated weekly age-standardised incidence rates (ASIRs) for working-age populations at the regional level of Germany’s 400 districts. Data covered nearly 2 years (March 2020 till December 2021), including four main waves of the pandemic. For each of the pandemic waves, we investigated regional differences in weekly ASIRs according to three regional labour market indicators: (1) employment rate, (2) employment by sector, and (3) capacity to work from home. We use spatial panel regression analysis, which incorporates geospatial information and accounts for regional clustering of infections.

**Results:**

For all four pandemic waves under study, we found that regions with higher proportions of people in employment had higher ASIRs and a steeper increase of infections during the waves. Further, the composition of the workforce mattered: rates were higher in regions with larger secondary sectors or if opportunities of working from home were comparatively low. Associations remained consistent after adjusting for potential confounders, including a proxy measure of regional vaccination progress.

**Conclusions:**

If further validated by studies using individual-level data, our study calls for increased intervention efforts to improve protective measures at the workplace, particularly among workers of the secondary sector with no opportunities to work from home. It also points to the necessity of strengthening work and employment as essential components of pandemic preparedness plans.

**Supplementary Information:**

The online version contains supplementary material available at 10.1186/s12879-022-07643-5.

## Background

After 2 years of the COVID-19 pandemic, the evidence on occupational determinants of SARS-CoV-2 infection risks is still limited. Among the existing studies, many focus on essential occupations and reveal that risks of infections are generally higher among health care workers, transport workers, teachers, child care workers, postal service workers, kitchen staff or workers in the logistic sector—but rather low for workers in the media sector (e.g., journalists), lawyers, scientist or workers in the financial sector [1–11]. These studies are instrumental as they help to describe infection risks for specific occupations. However, far-reaching conclusions on high-risk groups and corresponding policy implications remain limited, mainly because most studies focus on rather homogenous occupational groups without allowing for systematic comparisons of risks between different occupations. In addition, it is still not clear how findings based on individual-level data translate into regional differences of SARS-CoV-2 infections and corresponding incidence rates. Yet, knowledge on regional differences and the role of labour markets and their properties is instructive, as infections tend to cluster regionally and many decisions for pandemic intervention measures are made at regional levels [12]. Therefore, research based on individual data should be supplemented by ecological studies that examine levels of infections in relation to characteristics of regional labour markets—not as a substitute for individual studies (in case individual-level data is missing), but as a helpful supplement with immediate relevance for local and targeted policy decisions [13].

In fact, ecological studies on regional differences of COVID-19 have experienced increased attention during the pandemic [14, 15]. For example, studies from Germany [16–19], the UK [20], and the US [21, 22], suggest that levels of SARS-CoV-2 infections and COVID-19 mortality rates are comparatively higher in regions with high poverty rates or low income levels, or in regions that are generally socioeconomically disadvantaged. Interestingly, studies that also compare associations between different phases of the pandemic in Germany demonstrate that these socioeconomic differences are less pronounced (or even inversed) in the early first wave of the pandemic (with lower infection rates and mortality in more disadvantaged regions) [19, 23, 24]. Such an inverse association during the early phase of the pandemic was also found for the US [25] and France [26]. These findings highlight that socio-economic differences can also vary by different phases of the pandemic and that pandemic phases should be considered within the analyses. One possible explanation for these differences (and the reversion in the course of the pandemic) are different compositions of regional workforces and respective exposure to the virus. Higher infection rates at the beginning of the pandemic are possibly due to more employed people with international business travel, transregional commuting and more overall mobility (incl. holidays). In the course of the pandemic, though, these were exactly the occupational groups that were able to reduce their mobility with opportunities of working from home, while workers in less advantaged occupations were more exposed to the virus at their workplaces. Additionally, because of pre-existing health differences [27], workers in less advantaged occupations were then also more susceptible to an infection due to underlying health conditions.

Despite being mentioned as potential explanations, the role of regional labour markets and their properties, however, remains unclear. One exception is a recent study from Toronto that documents that infections and COVID-19 mortality rates (though for the overall population) are higher in neighbourhoods with a high proportion of people working in essential occupations [28]. Therefore, a comprehensive study of regional differences in levels of infections among the working-age population (those most likely to be affected by labour markets) that explicitly focus on regional labour markets and their properties (instead of socioeconomic factors) and takes changing patterns over time into account is still missing. It is the overall objective of this ecological study to fill this gap of knowledge for Germany, and to investigate how regional labour markets are related to levels of infections in the working population.

In the current ecological study, we rely on weekly nationwide data on notified laboratory-confirmed SARS-CoV-2 infections at the regional level for Germany (as an indicator of actual levels of infection in the population), and combine this data with regional labour market indicators to investigate differences in age-standardised incidence rates (ASIRs) of SARS-CoV-2 infections according to three labour market indicators. As previous studies show that associations between regional indicators and incidence rates vary by phases of the pandemic in Germany, we study associations separately for the first four main pandemic waves (ranging from March 2020 until December 2021). The three labour market indicators are: the overall extent of employment in a region, the composition of the workforce by economical job sectors, and existing opportunities of working from home. We also explicitly focus on the working-age population, as well as we apply a statistical approach (spatial panel analysis) which allows to address spatial autocorrelation—meaning that neighbouring regions are usually interrelated and not independent (see “Methods” for details).

## Methods

### Data sources

We combined the following data available at the German district level:

1. SARS-Cov-2 infections rates from the SurvStat@RKI 2.0 database,
2. geospatial information from the German Federal Agency for Cartography and Geodesy,
3. regional statistics on labour force participation as main exposures of interest, and
4. various regional population statistics (mainly as control variables in multivariable analyses).

### Weekly age-standardised SARS-CoV-2 incidence among working-age population

To assess regional levels of infections, we rely on nationwide regional data on weekly notified laboratory-confirmed SARS-CoV-2 infections from the SurvStat@RKI 2.0 database (data query on 16 March 2022) from the Robert-Koch-Institute (RKI). The RKI is the federal governmental institution that is responsible for national health surveillance of infectious diseases in Germany. For this, the RKI receives daily reports from the local health authorities on confirmed COVID-19 cases in accordance with the German Infection Protection Act. Because of the standardized and identical procedure of notification during the whole observation period of this study throughout Germany and widely established cost-free test systems (including public test stations) that immediately contact local health authorities in case of a positive PCR-test, data on notified infections must be seen as a close but relatively conservative approximation of the actual levels of infections. People with asymptomatic courses or with reduced likelihoods of using testing opportunities (e.g., reduced health literacy or difficult access) are possibly underrepresented, resulting in an estimate that likely represents the lower limit of the actual number of infections. The data is available for all 400 districts (German “Kreise” and “kreisfreien Städte”, the NUTS-3 level), which is the smallest area level available in the nationwide data on notified infections.

For the analyses, the covered time period ranged from 2 March 2020 (calendar week 10, as defined as start of the first wave [29, 30]) till 19 December 2021 (end of calendar week 51 in 2021). To calculate incidence rates (notified cases per 100,000 residents with same age), SurvStat@RKI uses population data (i.e. number of residents) from the Federal Statistical Office. To allow for meaningful comparisons of incidence rates between regions with different age compositions, we conducted direct age-standardisations and calculated age-standardised incidence rates (ASIRs). More specifically, we used age-specific incidence rates in 5 five-year intervals (from age 20 to age 64) and weighted each age group according to its distribution in the revised European Standard Population [31].

We used the whole observation period (for a description of trajectories) and distinguished between the following four main waves of the pandemic to account for possible variations when testing associations between labour market indicators and incidence rates: wave 1: week 10–20 (2 march 2020–17 may 2020); wave 2: week 40–week 8 (28 September 2020—28 February 2021); wave 3: week 9–week 23 (1 March 2021–13 June 2021); wave 4: week 31—week 51 (2 August 2021—26 December 2021). This distinction corresponds to the latest official German division of the pandemic phases of the Robert-Koch-Institute, as of March 2022 [29, 32].

### Geospatial information

Geospatial data came from the German Federal Agency for Cartography and Geodesy as a SHAPEfile [© GeoBasis-DE/BKG (2021)], with details at the district level for 401 districts. To enable linkage of geospatial data with incidence rates, we considered an administration reform of July 2021 (where two regions in Thuringia were merged into one) and merged the two respective region (or “polygons”) into one region using the “mergepoly” command in Stata [33]. Geospatial data both served as basis for maps and as essential part of the spatial regression models (see below for details).

### Regional labour market indicators

We included three indicators: (1) “employment rate”; (2) “employment by sector”, and (3) “capacity to work from home”.

Information on the regional employment rates came from the German Federal Agency for Work (“Bundesagentur für Arbeit”) [34]. It measures the proportion of working-age persons living in an area that is employed (not counting people who are unemployed or looking for a job) and refers to December 2019.

Employment by main sectors divided employment into the three broad economic sectors (primary, secondary and tertiary sector) in accordance to the European NACE systematic (“Nomenclature des activités économiques”). Specifically, we calculated the percentage of workers working in a sector of all workers. The primary sector covers occupation involved in basic production and the extraction of raw materials (i.e., agriculture, forestry, mining and fishery). The secondary sector includes manufacturing and construction. These are jobs that produce a finished, usable product or are involved in construction (e.g., machinist, food processing, construction worker). And the tertiary sector covers all occupations that distribute and sale goods, or provide services to other businesses or to final consumers (e.g., transport workers, health care workers, entertainment and media). We again used information from December 2019, provided by regional and federal offices of statistics [35].

Capacity to work from home consisted of a recently developed index that combines survey and administrative data [36]. In short, the index uses information on reported feasibility to work from home across different occupations (from the German BIBB/BAuA Employment Survey from 2018), and combines respective information with administrative data from the Federal Employment Agency on the frequency of different occupations by regions. This results in an index that quantifies the existing potential of working from home in each region as percentage of jobs in a region.

All labour market indicators were available for the 401 German districts before the administration reform mentioned above. We therefore calculate population weighted means of the two merged regions in case of “capacity to work from home”, or recalculate rates based on absolute values for the two other labour market indicators.

### Additional variables

We also included regional information on the following factors, mainly as potential confounders in multivariable analyses (after checking for possible multicollinearity between these variables): proportion of employees without professional qualification as percentage of all employees (as an indicator for the qualification level of the active workforce), proportion of female employees (to account for sex composition of the workforce), median salary income based on people living in the region (to consider the general income level of workers), district type based on four categories: “large city district”, “urban district”, “rural district with populated areas”, and “sparsely populated rural district” (to consider degree of urbanization), settlement density measured as number of residents per square kilometre in urban areas of settlement and transport space (accounting for residential density/proximity), average living space in square meter per accommodation (accounting for size of housing spaces), and border region (whether a district is a direct neighbour to another country). All variables were harmonised into 400 regions.

Details of each measure, including data sources and year of measurement are summarised in Table 1. Table 2 presents a correlation matrix of all measures used.

**Table 1:** Sample description for district level measures (400 regions): percentage (Col. %) or mean and standard deviation (SD), year and source

	Categories or range	Col % or mean (SD)	Year	Source
Employment rate	45.5–70.3	62.21 (4.15)	2019	Federal Agency for work
% of workers in 1st sector	0.0–8.4	1.93 (1.63)	2019	Federal Statistical Office of Germany
% of workers in 2nd sector	6.2–55.0	27.48 (9.24)	2019	Federal Statistical Office of Germany
% of workers in 3rd sector	44.4–93.8	70.59 (9.79)	2019	Federal Statistical Office of Germany
Capacity to work from home	47.3–65.1	54.02 (3.64)		[36]
% of employees without qualification	5.6–21.3	12.63 (3.14)	2019	Federal Agency for work
% of female employees	41.0–50.7	46.41 (1.81)	2019	Federal Agency for work
Average income in € (median)	2494.5–4668.6	3339.42 (394.33)		Federal Agency for work
District type	Large city district	16.75	2017	Federal Office for Building and Regional Planning
	Urban district	32.75		
	Rural district with populated areas	25.25		
	Sparsely populated rural district	25.25		
Settlement density (residents per km^2^)	612.0–6439.0	2170.02 (1059.10)	2018	Federal Statistical Office of Germany
Average living space (m^2^)	70.0–155.0	114.99 (18.24)	2017	Federal Statistical Office of Germany
Border region	Yes	89.25	2019	Federal Agency for Cartography and Geodesy
	No	10.75

**Table 2:** Correlations (Pearson’s) for all continuous district level measures

		1	2	3	4	5	6	7	8	9	10
1	Employment rate	1									
2	% of workers in 1^st^ sector	0.26	1								
3	% of workers in 2^nd^ sector	0.57	0.26	1							
4	% of workers in 3^rd^ sector	− 0.58	− 0.41	− 0.99	1						
5	Capacity to work from home	− 0.15	− 0.41	− 0.43	0.48	1					
6	% of employees without qualification	− 0.42	− 0.37	− 0.08	0.14	0.13	1				
7	% of female employees	− 0.15	0.05	− 0.41	0.38	0.14	− 0.53	1			
8	Average income in € (median)	− 0.08	− 0.27	− 0.02	0.06	0.72	0.46	− 0.38	1		
9	settlement density (residents per km^2^)	− 0.35	− 0.67	− 0.46	0.54	0.67	0.49	− 0.05	0.51	1	
10	Average living space (m^2^)	0.28	0.59	0.55	− 0.62	− 0.39	− 0.04	− 0.24	0.02	-0.67	1

### Analytical strategy

After a simple overview of all variables under study, we present the geographical distributions of ASIRs for the calendar weeks with the highest rate throughout Germany in each of the four waves, together with Moran’s test of residual correlation (to test for spatial autocorrelation) [37]. We then present trajectories for the entire observation period of ASIRs by different levels of regional labour market indicators. In that case, each labour market indicator was regrouped into “low”, “medium”, and “high” (based on tertiles) and we calculated mean scores of ASIRs by respective groups and calendar weeks.

For an in depth study of the associations between regional labour market indicators and regional ASIRs, we then estimated (separately for the four waves) spatial regression models for panel data including random effect for regions (i.e., random effects models) [14, 38]. In contrast to standard panel regression models (that assumes that neighbouring regions are independent), spatial models extend standard panel models by including geospatial information, and thereby, enable to account for spatial autocorrelation in different ways [14, 39]. Spatial models (for cross-sectional and panel data) are recently used extensively to evaluate geographical differences in COVID-19 and yield less biased estimates in case of spatial autocorrelation (see [14, 38, 39] for details). Spatial models principally follow two approaches to address spatial autocorrelation (and combinations and variations thereof): through a spatial lag model (also called spatial autoregressive (SAR) model) or through a spatial error model (SEM model). In short, the SEM reflects that neighbouring regions possibly share common characteristics (by including a spatial error component into the model), and the SAR model extends standard regressions by adding a “spatially lagged” dependent variable as predictor into the regression, thus, allowing that incidence rates of one regions can also affect incidence rates of neighbouring regions (spillover effects). Further extensions are a hybrid SAC model (combining both features of the SEM and SAR model), and a SLX model that includes spatially lagged explanatory variables. To allow for these extensions, spatial regressions require that geographical information is in a so called “spatial weighting matrix” that quantifies the distances between each pair of regions (resulting in a symmetrical 400 × 400 matrix in our case). In the present study, we defined the spatial matrix using a contiguity weighting matrix (first order, queen criterion) where direct neighbours can affect each other. To select the best fitting spatial model, we contrasted the AIC (Akaike Information Criterion) and the BIC (Bayesian Information Criterion) statistics of the four spatial Models named above to a standard linear model (SLM) for panel data, as well as we compared models based on likelihood ratio tests. Details are provided in the supplemental material (Additional file 1: Table S1). On that basis, we saw the that SLMs are likely to be misspecified (because of obvious autocorrelation) and opted for SEM Models, that revealed best model fits together with the hybrid SAC models (which rely on SEM). The SEM model is also the most widely used one in the literature.

As to the modelling strategy, we present estimates of two strategies. First, all models are estimated for each of the five labour market indicator separately (separate models, Table 3), and include the additional variables named above together with dummies for each calendar week. Second, we estimate models where labour market indicators are included simultaneously (with same adjustments, simultaneous models, Table 4). As such, the separate models allow to evaluate the total effect of each indicator on ASIRs, and the simultaneous models help to explore which of the indicator may still have a direct effect after conditioning for the remaining indicator, that is, the partial effect after removing contributions through other indicators [40]. In contrast to the descriptive analyses, the labour market indicators are each treated as continuous variables in the regression (avoiding loss of information). In doing so, we also tested for a possible non-linear relationship of the labour market indicators (by adding quadratic terms)—but these were not integrated in the final models due to absent additional explanatory power. In the Results section, we present estimated coefficients by wave for each of the labour market indicators (resulting in 20 models) together with confidence intervals (95%) and p-values. Finally, to summarise the main findings and to study if trajectories vary by labour market indicators, we re-estimate all models of Table 3 (separate models) with additional inclusion of interaction terms between calendar week and labour market indicators. On their basis, we predicted trajectories of incidence rate (i.e., “adjusted predictions at representative values”) for the cases of a high, medium and low value of each labour market indicator (based on Stata “margins” command [41]). Specifically, trajectories were predicted in case of a mean value (“medium”) and plus/minus one standard deviation (“high” and “low”) and are shown in Fig. 3. Results of the interaction tests (comparing models with and without interaction terms) are summarised in the supplemental material (Additional file 1: Table S3), presenting degrees of freedom (depending on the number of calendar weeks), the test statistics (Chi^2^) and corresponding p-values.

**Table 3:** Association between labour market indicators and age-standardised SARS-CoV-2 incidence rates for working-age population for different pandemic waves based on spatial error model for panel data (separate models): Coefficient (Coef.), confidence intervals (CI 95%), and p-values

	Wave 1			Wave 2			Wave 3			Wave 4		
	Coef	CI (95%)	p-value	Coef	CI (95%)	p-value	Coef	CI (95%)	p-value	Coef	CI (95%)	p-value
Employment rate	0.95	(0.52/1.37)	< 0.001	2.96	(1.79/4.13)	< 0.001	4.17	(3.14/5.20)	< 0.001	7.18	(5.12/9.24)	< 0.001
Employment by sectors (% in primary sector)	0.10	(− 1.15/1.35)	0.871	− 4.84	(− 8.24/− 1.44)	0.005	− 5.68	(− 8.79/− 2.57)	< 0.001	− 1.55	(− 7.73/4.64)	0.624
Employment by sectors (% in secondary sector)	0.41	(0.20/0.62)	< 0.001	2.16	(1.61/2.70)	< 0.001	2.68	(2.20/3.15)	< 0.001	4.85	(3.87/5.84)	< 0.001
Employment by sectors (% in tertiary sector)	− 0.43	(− 0.64/− 0.21)	< 0.001	− 2.07	(− 2.63/− 1.51)	< 0.001	− 2.59	(− 3.08/− 2.11)	< 0.001	− 4.84	(− 5.83/− 3.85)	< 0.001
Capacity to work from home	− 0.06	(− 1.00/0.87)	0.894	− 2.27	(− 4.84/0.30)	0.084	− 4.11	(− 6.45/− 1.78)	0.001	− 13.48	(− 17.87/− 9.10)	< 0.001

All models are calculated for each labour market indicator separately. Models are adjusted for proportion of employees without professional qualification, proportion of female employees, average income, district type, settlement density, average living space, and border region, as well as dummies are included for each calendar week

**Table 4:** Association between labour market indicators and age-standardised SARS-CoV-2 incidence rates for working-age population for different pandemic waves based on spatial error model for panel data (simultaneous models): Coefficient (Coef.), confidence intervals (CI 95%), and p-values

	Wave 1			Wave 2			Wave 3			Wave 4		
	Coef	CI (95%)	p-value	Coef	CI (95%)	p-value	Coef	CI (95%)	p-value	Coef	CI (95%)	p-value
Employment rate	0.71	(0.25/1.17)	0.003	1.51	(0.29/2.73)	0.015	2.51	(1.47/3.55)	< 0.001	4.87	(2.88/6.87)	< 0.001
Employment by sectors (% in primary sector)	0.44	(− 0.81/1.68)	0.490	− 3.02	(− 6.27/0.24)	0.069	− 3.39	(− 6.16/− 0.63)	0.016	0.25	(− 5.30/5.80)	0.930
Employment by sectors (% in secondary sector)	0.33	(0.09/0.57)	0.008	1.82	(1.19/2.46)	< 0.001	2.02	(1.48/2.56)	< 0.001	3.42	(2.36/4.47)	< 0.001
Employment by sectors (% in tertiary sector)^a^	–			–			–			–		
Capacity to work from home	0.39	(− 0.59/1.38)	0.435	0.24	(− 2.36/2.85)	0.856	− 1.27	(− 3.48/0.94)	0.261	-8.61	(− 12.95/− 4.27)	< 0.001

^a^Omitted because of collinearity

Labour market indicator are included simultaneously into the models. Models are adjusted for proportion of employees without professional qualification, proportion of female employees, average income, district type, settlement density, average living space, and border region, as well as dummies are included for each calendar week

As part of robustness checks, models were recalculated with alternative weighting matrices (i.e. inverse distance matrix). Additionally, in the case of wave 3 and wave 4 (where vaccination was possible), all models were rerun and additionally included a proxy measure on cumulative COVID-19 vaccination rates for each region (see Additional file 1 for details). All calculations, maps and graphs were produced with Stata 17.0, and we used the “spxtregress, re” procedure as part of the “sp” package for spatial panel analyses.

## Results

### Descriptive findings

**Fig. 1:**
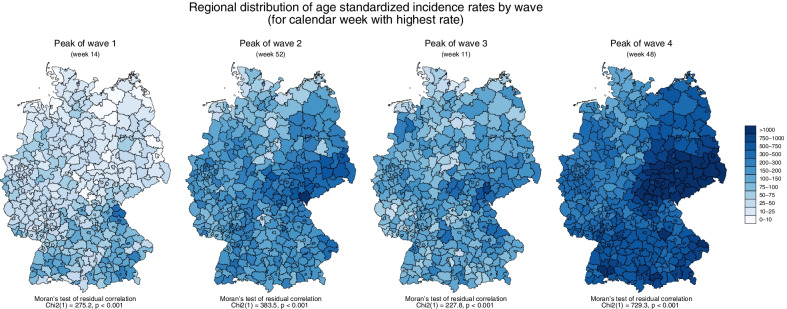
Regional distributions of weekly age standardised SARS-CoV-2 incidence rates by wave in Germany (for the calendar week with highest rate in Germany) and results of Moran’s test of residual correlation (spatial autocorrelation). Note. Shaded areas cover the four pandemic waves

Table 1 shows that the proportion of employed working-age persons in a region ranges from 45 to 70 percent, with a mean value of 62 percent. Also, we observe that most people either work in the secondary or the tertiary sector. In other words, regional labour markets are mainly divided between the secondary and tertiary sector. This also explains the very high correlation between the two later sectors in Table 2 (− 0.99). From Table 2 it is also worth noting that regions with a large tertiary sector have higher opportunities to work from home. Turning to the maps presented in Fig. 1 (and Moran’s test of residual correlation), there clearly are spatial autocorrelations in ASIRs in all four waves, with clustering of high rates during the first wave in Southern Germany (specifically Bavaria) and a clustering of high rates in Eastern Germany (specifically Thuringia and Saxony) during the remaining waves.

**Fig. 2:**
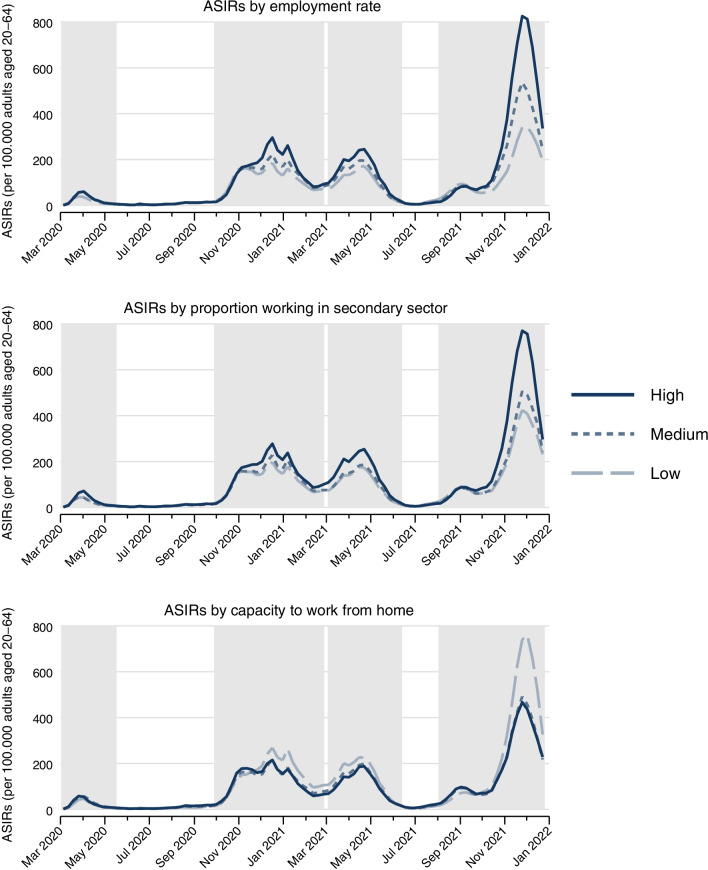
Trajectories of weekly age-standardised SARS-CoV-2 incidence rates (ASIRs) for working-age population (aged 20–64 years) by levels of regional labour market indicators (based on tertiles) in Germany

Figure 2 gives a first answer on how ASIRs for working-age populations differ by regional labour market indicators. The shaded areas cover the four pandemic waves and—in the case of employment sectors—the figure focuses on the secondary sector (because the primary sector is negligible and because findings for the third sector are complementary). With except of wave 1, rates are highest in regions with a high employment rate. Also, regions with a high proportion of people working in the secondary sector (and vice-versa with a less established third sector) or with low capacities to work from home have generally higher ASIRs—at all studied phases of the pandemic.

### Results of spatial regression models

Estimates of main analyses are presented in Tables 3 and 4, with four findings worth noting: First, employment rates and incidence rates are positively associated in all four waves, with lowest estimates in wave 1 (where weekly incidences rates are generally lower). Second, turning to employment sectors, regions with a pronounced secondary sector have higher ASIRs—again specifically in wave 2 to 4. Conversely, regions with a pronounced tertiary sector reveal lower rates. Third, from wave 2 to 4 we see that higher capacities to work from home are increasingly related to lower incidence rates. Fourth, when comparing results between Tables 3 and 4, estimates are generally attenuated but remained significant in most cases, even after conditioning for the remaining career characteristics (with the only exception of capacity to work from home in wave 3), thus suggesting that the indicators are independently related to ASIRs. In addition, this indicates that capacity to work from home could be an intermediate factor (partial mediation) linking employment rate and the employment sector with ASIRs, as well as the employment sector partly acts a confounder in the case of capacity to work from home (where a pronounced secondary sector affects both capacity to work from home and ASIRs). In sum, our findings confirm descriptive results, even after adjusting for various factors (incl. settlement density, district type, border region, level of qualification, proportion of female employees) and considering spatial autocorrelation, with p-values providing evidence against the null-hypothesis for all the reported associations. Findings for wave 3 and wave 4 also remain consistent in sensitivity analyses additionally adjusted for a proxy measure of vaccination rates (see Additional file 1: Table S2 for details).

**Fig. 3:**
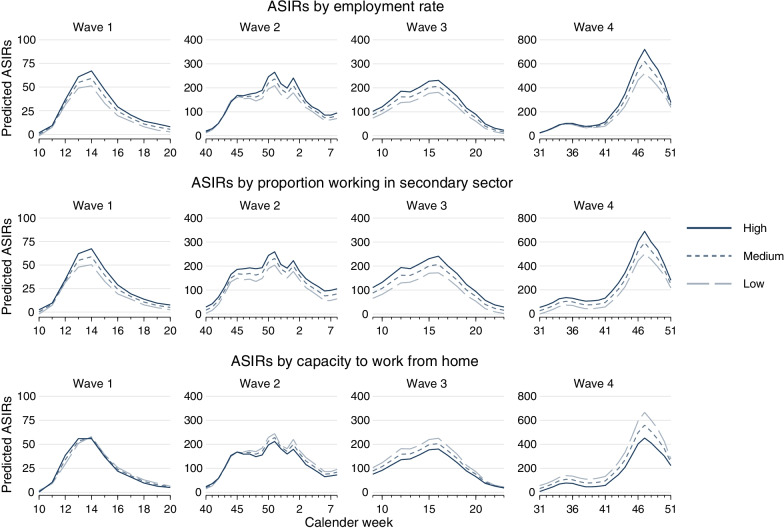
Predicted age-standardised SARS-CoV-2 incidence rates (ASIRs) for working age population (aged 20–64 years) at given levels of labour market indicators (mean + − 1 SD) for different pandemic waves based on spatial error model for panel data (same adjustments as in Table 3)

To summarize the main findings, Fig. 3 presents the predicted ASIRs at given levels of labour market indicators (mean +—1 SD) for each calendar week under study (adjusted for same covariates as in Table 3). Again, we clearly see that rates are particularly high for regions with higher rates, with a high proportion of people working in the secondary sector, or with low capacities to work from home. Furthermore, for these latter regions, the increase in incidence rates (beside the overall level) appears generally steeper—a result that is further supported by the fact that interactions between labour market indicators and calendar were all significant with p-values < 0.001 (see Additional file 1: Table S3 for details).

## Discussion

This ecological study provides evidence that regional labour markets and their properties are related to regional patterns of SARS-CoV-2 infections in the working-age population in Germany. In detail, for all four phases under study we find that regions with higher proportions of people in employment have generally higher weekly age-standardised incidence rates, and that regions where more people work in the secondary sector or with low capacities to work from home (mainly in waves 3 and 4 though) have higher rates as well. Furthermore, findings indicate that these latter regions also experience a steeper increase of infection rates in the course of the four waves under study. Findings are based on spatial models that account for spatial autocorrelation and remained stable after adjusting for potential confounders at the regional level. And—in cases of wave 3 and wave 4—the reported findings were also found when additionally adjusting for a proxy measure of vaccination progress.

Overall, the observed associations are in line with previous studies, specifically ecological studies that investigate socioeconomic deprivation in conjunction with SARS-CoV-2 infection rates or COVID-19 mortality [23, 42]. Yet, by focussing on working-age populations and conducting refined spatial panel analyses of trajectories of weekly age-standardised incidence rates that consider both regional clustering and potential regional confounders, we provide evidence that adds to existing research in at least two ways: First, by including an indicator that measures the general amount of employment at the regional level, we highlight that work and employment could be key factors for infection transmissions in a region, and thus, that the workplace may be an important entry point for interventions. Second, the finding of higher infections rates in regions with a large secondary sector (or conversely a small tertiary sector) adds to current knowledge that is either limited to smaller geographical areas [28], or relies on studies that use cumulative infection rates across an extended observation period as outcome without focussing on labour market factors [43, 44]. Sure, the considered occupations of the secondary sector (i.e., all occupations involved in the production or the construction of goods) must be considered as heterogeneous in our case, clearly asking for refined analyses to investigate if certain occupations within the sectors are driving the varying incidence rates. But our findings give good reasons to believe that people who work in regions where more people work in the secondary sector are generally more likely to be exposed to the virus (at least for the studied periods of the pandemic). This is also supported by studies relying on individual data that show that essential workers in the secondary sector (e.g., food production) have higher rates and that many jobs of the tertiary sector (e.g., financial services or media) have lower rates (with the exception of health care workers) [2, 8]. On the one hand, we may speculate that protective measures in the secondary sector (e.g., social distancing or use of face masks) are less established and less effective (e.g., when working in a large manufacturing hall compared with office work). Also, the number of co-workers in close proximity at work is possibly higher compared with office work. On the other hand, though, our results suggest that opportunities of working from home, including the possibility to reduce work-related mobility (e.g., public transport to the workplace), are much smaller in jobs of the secondary sector. The latter idea is also supported by our finding of a negative correlation between size of the secondary sector and capacity to work from home, as well as in models where labour market indicators are included simultaneously. The named aspects (i.e., transmission risks, mitigation measures, work from home) are also important components of recent efforts to estimate potential SARS-CoV-2 infection risks and to develop a respective job exposure matrix [45, 46]. Another reason, though, may be that the sectors were differently affected by closures as part of the non-pharmaceutical interventions implemented to contain the pandemic. In fact, while large parts of the tertiary sectors were affected through closures of several businesses (the gastronomy, cultural institutions, or shops), many industries of the secondary sectors remained open.

On a more general note, our study illustrates the necessity of extending the growing evidence on socioeconomic differences in infection risks to factors that are considered as potential explanations. Future ecological studies also need to focus on other potential explanations of socioeconomic differences, such as pre-existing health conditions [27], air pollution [47, 48] (as two potential reasons for greater vulnerability), or on measures capturing different access and use of medical care in a region (incl. adherence to NPI and vaccination coverage) [49]. Here, the included measure of vaccination rate as part of our sensitivity analyses must clearly be seen as a preliminary measure that deserves more methodological refinements (assuring that we know where vaccinated people live) and more analyses.

The study has several limitations: First, we again need to consider that work and employment are possibly one—though it is not the only factor that may explain regional variations of infection rates, and other factors equally deserve attention in future studies to understand varying infection risks [50, 51]. Beside those just named above, these may also be more general aspects not necessarily related to socioeconomic deprivation, such as meteorological information (e.g., number of raining days or average temperature), sanitation or hygiene, public transport systems or policy interventions at the regional level. Second, albeit we maintain that ecological studies are instrumental, and well-suited to supplement individual data (because of the direct relevance for potential interventions), we need to be very careful when drawing conclusion from the regional to the individual level. At this point, we need to remember that—albeit being the smallest level available—our study relies on rather large areas. We therefore must consider the risk of an ecological fallacy including potential heterogeneity of workers within regions. To be clear, we cannot guarantee that those who are employed in the secondary sector of a region are also those who are infected. Future extensions of this study, therefore, should study if our findings are confirmed at a more granular regional scale (with possibly more pronounced associations), as well as on the basis of individual-level data. Another limitation relates to testing strategies in the regions. While our study is based on official data on notified laboratory-confirmed infections based on widely accessible testing opportunities and identical notification procedures throughout Germany, we still may ask if some occupational groups could be more likely to be tested in some regions than others (and thus to be detected and notified as cases). Yet, because testing opportunities (i.e., antigen rapid tests with subsequent PCR-test in case of positive result) were free of charge throughout the whole observation period of this study, and because respective policy changes are decided at the federal level for all regions (irrespective of the workforce composition of a region), it is unlikely that the found differences by regions are affected by changes of testing strategies during the observation period. In a similar way, it is known that health seeking behaviour varies by occupation [52], meaning that some infections are possibly more likely to remain undetected for some occupational groups. A recent German study, for example, compared nationwide findings from a SARS-CoV-2 seroepidemiological study with data on notified infections and estimated that up till 45% of SARS-CoV-2 infections remained undetected [53], with slightly higher values in socioeconomically disadvantaged regions. Along these lines, we may speculate if our study underestimates the association with ASIRs in the cases of the secondary sector and low capacities to work from home, as these are possibly jobs with more people working in less advantaged occupations.

Another limitation relates to the generalisation of results. Albeit our study covers nearly 2 years of the pandemic and distinguishes four periods, insights into subsequent waves with other dominant virus variants are not possible. Results also need to be replicated for other countries. Finally, although our study firstly allows to compare regional variations in infection rates focusing on working-age populations, we still need to question if the observed differences for working-age populations are also translated into differences at the population level. Because infections are likely to be transmitted within families, and because the overall regional disparities correspond to what is observed for the general population [16, 23], there are good reasons to think that this is the case.

## Conclusions

In conclusion, our study extends current knowledge, by analysing regional variations of SARS-CoV-2 infection risks across four waves of the pandemic by regional labour markets and their properties. This underlines the importance of work and employment as key domains and places for transmission risks. In doing so, it points to the necessity of strengthening these factors as essential component of pandemic preparedness plans and to amend workplace interventions, particularly among workers of the secondary sector without opportunities of working from home.

## Supplementary Information

**Additional file 1.** Supplementary tables.

## Data Availability

All data of this study is freely available without need of administrative permissions. Access to data for weekly incidence rates of laboratory-confirmed SARS-CoV-2 infections exists through the SurvStat@RKI 2.0 database of the Robert Koch Institute, and through official administrative sources for regional indicators of work and employment and population statistics. The code of data management and data analyses are available upon request from the corresponding author.

## Abbreviations

SARS-CoV-2: Severe acute respiratory syndrome coronavirus type 2
ASIRs: Age-standardised incidence rates
COVID-19: Coronavirus disease 2019
RKI: Robert-Koch-Institute
NUTS: Nomenclature of territorial units for statistics
NACE: Nomenclature des activités économiques
BKG: Bundesamt für Kartographie und Geodäsie
BIBB: Bundesinstitut für Berufsbildung
BAuA: Bundesanstalt für Arbeitsschutz und Arbeitsmedizin
SAR model: Spatial autoregressive model
SEM model: Spatial error model
SLX model: Spatially-lagged X model
AIC: Akaike information criterion
BIC: Bayesian information criterion
SLM: Standard linear model
SD: Standard deviation
NPI: Non-pharmaceutical interventions
PCR-test: Polymerase chain reaction test

## References

[1] Galanis P, Vraka I, Fragkou D, Bilali A, Kaitelidou D (2020). Seroprevalence of SARS-CoV-2 antibodies and associated factors in health care workers: a systematic review and meta-analysis. J Hosp Infect.

[2] Mutambudzi M, Niedwiedz C, Macdonald EB (2020). Occupation and risk of severe COVID-19: prospective cohort study of 120 075 UK Biobank participants. Occup Environ Med.

[3] Magnusson K, Nygård K, Methi F, Vold L, Telle K. Occupational risk of COVID-19 in the 1st vs 2nd wave of infection. MedRxiv. 2021:2020.10. 29.20220426.

[4] Chadeau-Hyam M, Bodinier B, Elliott J (2020). Risk factors for positive and negative COVID-19 tests: a cautious and in-depth analysis of UK biobank data. Int J Epidemiol.

[5] Möhner M, Wolik A (2020). Berufs-und branchenbezogene Unterschiede im COVID-19-Risiko in Deutschland. Dtsch Arztebl Int.

[6] AOK Nordwest. Krankschreibungs-Analyse der AOK Nordost für Berlin: Erzieherinnen haben das höchste Corona-Infektionsrisiko. https://www.aok.de/pk/nordost/inhalt/krankschreibungs-analyse-der-aok-nordost-fuer-berlin/. 2021.

[7] Rimmer A (2020). Covid-19: Two thirds of healthcare workers who have died were from ethnic minorities. BMJ.

[8] Stringhini S, Zaballa M-E, Pullen N (2021). Large variation in anti-SARS-CoV-2 antibody prevalence among essential workers in Geneva, Switzerland. Nat Commun.

[9] Laajaj R, De Los RC, Sarmiento-Barbieri I (2021). COVID-19 spread, detection, and dynamics in Bogota, Colombia. Nat Commun.

[10] Nafilyan V, Pawelek P, Ayoubkhani D (2021). Occupation and COVID-19 mortality in England: a national linked data study of 14.3 million adults. Occup Environ Med.

[11] Wachtler B, Neuhauser H, Haller S (2021). The risk of infection with SARS-CoV-2 among healthcare workers during the pandemic. Dtsch Arztebl International.

[12] Bambra C, Riordan R, Ford J, Matthews F (2020). The COVID-19 pandemic and health inequalities. J Epidemiol Community Health.

[13] Schwartz S (1994). The fallacy of the ecological fallacy: the potential misuse of a concept and the consequences. Am J Public Health.

[14] Guliyev H (2020). Determining the spatial effects of COVID-19 using the spatial panel data model. Spatial Statistics.

[15] Björk J, Modig K, Kahn F, Ahlbom A (2021). Revival of ecological studies during the COVID-19 pandemic. Eur J Epidemiol.

[16] Dragano N, Hoebel J, Wachtler B, Diercke M, Lunau T, Wahrendorf M. Soziale Ungleichheit in der regionalen Ausbreitung von SARS-CoV-2. Bundesgesundheitsblatt-Gesundheitsforschung-Gesundheitsschutz. 2021:1–9. 10.1007/s00103-021-03387-w PMC8298974 34297163

[17] Hoebel J, Michalski N, Diercke M (2021). Emerging socioeconomic disparities in COVID-19–related deaths during the second pandemic wave in Germany. Int J Infect Dis.

[18] Plümper T, Neumayer E (2020). The pandemic predominantly hits poor neighbourhoods? SARS-CoV-2 infections and COVID-19 fatalities in German districts. Eur J Public Health.

[19] Wachtler B, Michalski N, Nowossadeck E (2020). Socioeconomic inequalities in the risk of SARS-CoV-2 infection—First results from an analysis of surveillance data from Germany. J Health Monitoring.

[20] ONS. Deaths involving COVID-19 by local area and socioeconomic deprivation: deaths occurring between 1 March and 17 April 2020. London: Office for National Statistics; 2020.

[21] Whittle RS, Diaz-Artiles A (2020). An ecological study of socioeconomic predictors in detection of COVID-19 cases across neighborhoods in New York City. BMC Med.

[22] Abedi V, Olulana O, Avula V (2021). Racial, economic, and health inequality and COVID-19 infection in the United States. J Racial Ethn Health Disparities.

[23] Hoebel J, Michalski N, Wachtler B (2021). Socioeconomic differences in the risk of infection during the second SARS-CoV-2 wave in Germany. Dtsch Arztebl Int.

[24] Wachtler B, Michalski N, Nowossadeck E (2020). Socioeconomic inequalities and COVID-19: a review of the current international literature. J Health Monitoring..

[25] Finch WH, Hernández Finch ME (2020). Poverty and COVID-19: rates of incidence and deaths in the United States during the first 10 weeks of the pandemic. Front Sociol.

[26] Ginsburgh V, Magerman G, Natali I (2021). COVID-19 and the role of inequality in French regional departments. Eur J Health Econ.

[27] Marmot M, Allen J (2020). COVID-19: exposing and amplifying inequalities. J Epidemiol Community Health.

[28] Rao A, Ma H, Moloney G (2021). A disproportionate epidemic: COVID-19 cases and deaths among essential workers in Toronto, Canada. Ann Epidemiol.

[29] Schilling J, Tolksdorf K, Marquis A (2021). Die verschiedenen Phasen der COVID-19-Pandemie in Deutschland: Eine deskriptive Analyse von Januar 2020 bis Februar 2021. Bundesgesundheitsblatt Gesundheitsforschung Gesundheitsschutz.

[30] Tolksdorf K, Buda S, Schilling J. Aktualisierung zur Retrospektiven Phaseneinteilung der COVID-19-Pandemie in Deutschland. 2021.

[31] Eurostat. Revision of the European Standard Population. Report of Eurostat's task force. Luxembourg: Publications Office of the European Union; 2013.

[32] Schilling J, Buda S, Tolksdorf K (2022). Zweite Aktualisierung der Retrospektiven Phaseneinteilung der COVID-19- Pandemie in Deutschland. Epidemiologisches Bulletin.

[33] Picard R, Stepner M. MERGEPOLY: Stata module to merge adjacent polygons from a shapefile: Boston College Department of Economics; 2015.

[34] Bundesagentur für Arbeit. Beschäftigungsquoten (Jahreszahlen und Zeitreihen). Nürnberg: Bundesagentur für Arbeit, Statistik; 2020.

[35] Statistische Ämter des Bundes und der Länder. Erwerbstätigenrechnung—Erwerbstätige in den kreisfreien Städten und Landkreisen der Bundesrepublik Deutschland 1991 bis 2019. Reihe 2, Band 1. Wiesbaden: Arbeitskreis. „Erwerbstätigenrechnung des Bundes und der Länder”; 2021.

[36] Alipour J-V, Falck O, Schüller S. Germany’s capacities to work from home. Bonn: IZA DISCUSSION PAPER SERIES; 2020.

[37] Drukker DM, Prucha IR (2013). On the I 2( q ) test statistic for spatial dependence: finite sample standardization and properties. Spat Econ Anal.

[38] Elhorst JP (2014). Spatial econometrics from cross-sectional data to spatial panels.

[39] Darmofal D (2015). Spatial analysis for the social sciences.

[40] Westreich D, Greenland S (2013). The table 2 fallacy: presenting and interpreting confounder and modifier coefficients. Am J Epidemiol.

[41] Williams R (2012). Using the margins command to estimate and interpret adjusted predictions and marginal effects. Stata J.

[42] Wachtler B, Michalski N, Nowossadeck E (2020). Sozioökonomische Ungleichheit im Infektionsrisiko mit SARS-CoV-2—Erste Ergebnisse einer Analyse der Meldedaten für Deutschland. J Health Monitoring.

[43] Ehlert A (2021). The socio-economic determinants of COVID-19: a spatial analysis of German county level data. Socioecon Plann Sci.

[44] Alipour J-V, Fadinger H, Schymik J. My home is my castle: the benefits of working from home during a pandemic crisis. Evidence from Germany: ifo Working Paper2020.

[45] Oude Hengel K, Burdorf A, Pronk A (2021). Exposure to a SARS-CoV-2 infection at work: development of an international job exposure matrix (COVID-19-JEM). Scand J Work Environ Health.

[46] Zhang M (2021). Estimation of differential occupational risk of COVID-19 by comparing risk factors with case data by occupational group. Am J Ind Med.

[47] Fairburn J, Schule SA, Dreger S, Karla Hilz L, Bolte G (2019). Social inequalities in exposure to ambient air pollution: a systematic review in the WHO European region. Int J Environ Res Public Health.

[48] Marquès M, Domingo JL (2022). Positive association between outdoor air pollution and the incidence and severity of COVID-19. A review of the recent scientific evidences. Environ Res.

[49] Gollwitzer A, Martel C, Brady WJ (2020). Partisan differences in physical distancing are linked to health outcomes during the COVID-19 pandemic. Nat Hum Behav.

[50] Quinn SC, Kumar S (2014). Health inequalities and infectious disease epidemics: a challenge for global health security. Biosecur Bioterror.

[51] Bambra C (2022). Pandemic inequalities: emerging infectious diseases and health equity. Int J Equity Health.

[52] Biggerstaff M, Jhung MA, Reed C, Fry AM, Balluz L, Finelli L (2014). Influenza-like illness, the time to seek healthcare, and influenza antiviral receipt during the 2010–2011 influenza season-United States. J Infect Dis.

[53] Neuhauser H, Rosario AS, Butschalowsky H (2021). Germany’s low SARS-CoV-2 seroprevalence confirms effective containment in 2020: Results of the nationwide RKI-SOEP study. medRxiv.

