# Supplementary material for: Regional disparities in SARS-CoV-2 infections by labour market indicators: a spatial panel analysis using nationwide German data on notified infections
Source: BMC Infect Dis. 2022 Jul 30;22:661. doi: 10.1186/s12879-022-07643-5 (PMC9338475; doi:10.1186/s12879-022-07643-5)
Supplement: Supplementary file 1 — Additional file 1. Supplementary tables. [file 12879_2022_7643_MOESM1_ESM.docx]

**Supplementary material**

Table S1. Model fit comparisons of standard linear panel models (SLM), spatial standard error models (SEM), spatial autoregressive models (SAR), hybrid model (SAC), and spatially-lagged X model (SLX) for each labour market indicator and pandemic wave: AIC (Akaike Information Criterion), BIC (Bayesian Information Criterion), and chi² (contrast to SLM)

|  |  | | **Wave 1** | | |  | |  |  | | **Wave 2** | | |  | |  |  | | **Wave 3** | | |  | |  |  | | **Wave 4** | | |  | |
| --- | --- | --- | --- | --- | --- | --- | --- | --- | --- | --- | --- | --- | --- | --- | --- | --- | --- | --- | --- | --- | --- | --- | --- | --- | --- | --- | --- | --- | --- | --- | --- |
|  | SLM | SEM | | SAR | SAC | | SLX |  | SLM | SEM | | SAR | SAC | | SLX |  | SLM | SEM | | SAR | SAC | | SLX |  | SLM | SEM | | SAR | SAC | | SLX |
|  |  |  | |  |  | |  |  |  |  | |  |  | |  |  |  |  | |  |  | |  |  |  |  | |  |  | |  |
| *Employment rate* |  |  | |  |  | |  |  |  |  | |  |  | |  |  |  |  | |  |  | |  |  |  |  | |  |  | |  |
| AIC | 39791 | 38694 | | 38988 | 38696 | | 39779 |  | 98895 | 95501 | | 96190 | 95503 | | 98897 |  | 63905 | 62568 | | 62940 | 62557 | | 63907 |  | 110449 | 102502 | | 104506 | 102495 | | 110445 |
| BIC | 39938 | 38848 | | 39141 | 38856 | | 39933 |  | 99136 | 95749 | | 96437 | 95758 | | 99145 |  | 64086 | 62756 | | 63127 | 62752 | | 64094 |  | 110682 | 102741 | | 104745 | 102741 | | 110684 |
| LR test chi² (contrast to SLM) |  | 1099.0 | | 805.5 | 1099.2 | | 13.9 |  |  | 3396.4 | | 2707.8 | 3396.7 | | 0.0 |  |  | 1339.1 | | 967.4 | 1351.8 | | 0.4 |  |  | 7949.6 | | 5945.8 | 7958.7 | | 6.8 |
|  |  |  | |  |  | |  |  |  |  | |  |  | |  |  |  |  | |  |  | |  |  |  |  | |  |  | |  |
| *Employment by sectors (% in primary sector)* |  |  | |  |  | |  |  |  |  | |  |  | |  |  |  |  | |  |  | |  |  |  |  | |  |  | |  |
| AIC | 39819 | 38713 | | 38998 | 38714 | | 39816 |  | 98914 | 95517 | | 96185 | 95518 | | 98908 |  | 63952 | 62615 | | 62954 | 62598 | | 63951 |  | 110517 | 102548 | | 104513 | 102539 | | 110513 |
| BIC | 39966 | 38866 | | 39151 | 38874 | | 39969 |  | 99155 | 95765 | | 96432 | 95773 | | 99156 |  | 64133 | 62803 | | 63142 | 62792 | | 64139 |  | 110749 | 102787 | | 104752 | 102786 | | 110753 |
| LR test chi² (contrast to SLM) |  | 1107.9 | | 823.1 | 1108.3 | | 5.0 |  |  | 3398.9 | | 2731.8 | 3400.0 | | 8.5 |  |  | 1338.8 | | 999.7 | 1357.8 | | 2.9 |  |  | 7971.2 | | 6006.1 | 7981.4 | | 5.3 |
|  |  |  | |  |  | |  |  |  |  | |  |  | |  |  |  |  | |  |  | |  |  |  |  | |  |  | |  |
| *Employment by sectors (% in secondary sector)* |  |  | |  |  | |  |  |  |  | |  |  | |  |  |  |  | |  |  | |  |  |  |  | |  |  | |  |
| AIC | 39789 | 38698 | | 38986 | 38699 | | 39790 |  | 98859 | 95468 | | 96173 | 95470 | | 98859 |  | 63840 | 62515 | | 62892 | 62501 | | 63829 |  | 110379 | 102459 | | 104483 | 102451 | | 110380 |
| BIC | 39936 | 38851 | | 39139 | 38859 | | 39944 |  | 99100 | 95716 | | 96421 | 95725 | | 99107 |  | 64021 | 62703 | | 63079 | 62696 | | 64017 |  | 110612 | 102698 | | 104722 | 102697 | | 110619 |
| LR test chi² (contrast to SLM) |  | 1093.1 | | 804.9 | 1093.6 | | 0.7 |  |  | 3392.9 | | 2687.7 | 3393.2 | | 1.7 |  |  | 1326.7 | | 950.0 | 1342.5 | | 12.3 |  |  | 7922.9 | | 5898.7 | 7932.4 | | 1.5 |
|  |  |  | |  |  | |  |  |  |  | |  |  | |  |  |  |  | |  |  | |  |  |  |  | |  |  | |  |
| *Employment by sectors (% in tertiary sector)* |  |  | |  |  | |  |  |  |  | |  |  | |  |  |  |  | |  |  | |  |  |  |  | |  |  | |  |
| AIC | 39788 | 38697 | | 38986 | 38699 | | 39771 |  | 98865 | 95474 | | 96177 | 95476 | | 98867 |  | 63853 | 62526 | | 62902 | 62513 | | 63855 |  | 110379 | 102460 | | 104485 | 102452 | | 110363 |
| BIC | 39935 | 38851 | | 39140 | 38859 | | 39924 |  | 99106 | 95722 | | 96425 | 95731 | | 99115 |  | 64034 | 62714 | | 63090 | 62707 | | 64042 |  | 110612 | 102699 | | 104724 | 102699 | | 110602 |
| LR test chi² (contrast to SLM) |  | 1093.0 | | 804.1 | 1093.5 | | 19.3 |  |  | 3393.1 | | 2690.3 | 3393.4 | | 0.1 |  |  | 1328.8 | | 952.8 | 1343.9 | | 0.0 |  |  | 7921.9 | | 5896.6 | 7931.0 | | 18.3 |
|  |  |  | |  |  | |  |  |  |  | |  |  | |  |  |  |  | |  |  | |  |  |  |  | |  |  | |  |
| *Capacity to work from home* |  |  | |  |  | |  |  |  |  | |  |  | |  |  |  |  | |  |  | |  |  |  |  | |  |  | |  |
| AIC | 39819 | 38713 | | 38997 | 38714 | | 39811 |  | 98922 | 95522 | | 96185 | 95523 | | 98924 |  | 63958 | 62616 | | 62955 | 62600 | | 63958 |  | 110495 | 102513 | | 104487 | 102504 | | 110495 |
| BIC | 39966 | 38866 | | 39150 | 38874 | | 39964 |  | 99163 | 95770 | | 96433 | 95778 | | 99172 |  | 64139 | 62804 | | 63142 | 62794 | | 64145 |  | 110727 | 102752 | | 104726 | 102750 | | 110734 |
| LR test chi² (contrast to SLM) |  | 1108.0 | | 823.7 | 1108.4 | | 9.9 |  |  | 3402.2 | | 2739.1 | 3403.3 | | 0.5 |  |  | 1343.7 | | 1005.3 | 1362.5 | | 2.2 |  |  | 7983.9 | | 6010.0 | 7995.0 | | 2.0 |
|  |  |  | |  |  | |  |  |  |  | |  |  | |  |  |  |  | |  |  | |  |  |  |  | |  |  | |  |

Note. All models are calculated for each labour market indicator separately. Models are adjusted for proportion of employees without professional qualification, proportion of female employees,
average income, district type, settlement density, average living space, and border region, as well as dummies are included for each calendar week.

Table S2. Association between labour market indicators and age-standardized incidence rates for working aged populations for waves 3 and 4 based on spatial error models for panel data: Coefficient (Coef.), confidence intervals (CI 95%), and p-values (additional adjustments for a proxy of vaccination rates ^a^)

|  |  | **Wave 3** | | |  | **Wave 4** | | |
| --- | --- | --- | --- | --- | --- | --- | --- | --- |
|  |  | Coef. | CI (95%) | p-value |  | Coef. | CI (95%) | p-value |
|  |  |  |  |  |  |  |  |  |
| Employment rate |  | 4.14 | (3.11/5.18) | <0.001 |  | 7.17 | (5.11/9.23) | <0.001 |
|  |  |  |  |  |  |  |  |  |
| Employment by sectors (% in primary sector) |  | -5.84 | (-8.96/-2.72) | <0.001 |  | -2.34 | (-8.57/3.89) | 0.462 |
|  |  |  |  |  |  |  |  |  |
| Employment by sectors (% in secondary sector) |  | 2.66 | (2.18/3.14) | <0.001 |  | 4.72 | (3.73/5.72) | <0.001 |
|  |  |  |  |  |  |  |  |  |
| Employment by sectors (% in tertiary sector) |  | -2.57 | (-3.06/-2.08) | <0.001 |  | -4.70 | (-5.70/-3.71) | <0.001 |
|  |  |  |  |  |  |  |  |  |
| Capacity to work from home |  | -4.07 | (-6.41/-1.73) | 0.001 |  | -13.12 | (-17.53/-8.70) | <0.001 |
|  |  |  |  |  |  |  |  |  |

Note. All models are calculated for each labour market indicator separately. Models are adjusted for proportion of employees without professional qualification, proportion of female employees,
average income, district type, settlement density, average living space, and boarder region, as well as dummies are included for each calendar week.

^a^ As a proxy measures of regional vaccination rates, the sensitivity analysis uses information from the Digital vaccination rate monitoring from the Robert-Koch-Institute (German: “Digitales Impfquoten Monitoring”, accessed 10 January 2022). The monitoring contains daily information from January 2021 onwards on number of vaccinations (classified as “first”, “second”, or “booster” shot) at a regional level. Importantly, the monitoring contains information on the region where the vaccination occurred (but not where the vaccinated person lives). Thus, it is not guaranteed that vaccinated people also live in the region. For the analyses, we first calculated for each calendar week the proportion of vaccinated persons of the total population in the region that received full vaccinations (two shots), and then calculated the cumulative rates with a two-week delay (corresponding to the assumed time frame needed to achieve disease protection).

Table S3. Results of tests of significant interactions between calendar week and labour market indicators based on
spatial error models for panel data: degrees of freedom (df), Chi² and p-values.

|  | **Wave 1** | | |  | **Wave 2** | | |  | **Wave 3** | | |  | **Wave 4** | | |
| --- | --- | --- | --- | --- | --- | --- | --- | --- | --- | --- | --- | --- | --- | --- | --- |
|  | (df) |  | Chi2 (p-value) |  | (df) |  | Chi2 (p-value) |  | (df) |  | Chi2 (p-value) |  | (df) |  | Chi2 (p-value) |
|  |  |  |  |  |  |  |  |  |  |  |  |  |  |  |  |
| Employment rate X Calendar week | 10 |  | 39.45 (<0.001) |  | 21 |  | 192.55 (<0.001) |  | 14 |  | 86.39 (<0.001) |  | 20 |  | 509.20 (<0.001) |
|  |  |  |  |  |  |  |  |  |  |  |  |  |  |  |  |
| Employment by sectors (% in primary sector) X Calendar week | 10 |  | 13.09 (0.219) |  | 21 |  | 37.50 (0.015) |  | 14 |  | 29.31 (0.010) |  | 20 |  | 118.61 (<0.001) |
|  |  |  |  |  |  |  |  |  |  |  |  |  |  |  |  |
| Employment by sectors (% in secondary sector) X Calendar week | 10 |  | 56.09 (<0.001) |  | 21 |  | 40.87 (0.006) |  | 14 |  | 101.19 (<0.001) |  | 20 |  | 228.58 (<0.001) |
|  |  |  |  |  |  |  |  |  |  |  |  |  |  |  |  |
| Employment by sectors (% in tertiary sector) X Calendar week | 10 |  | 56.34 (<0.001) |  | 21 |  | 34.42 (0.033) |  | 14 |  | 79.93 (<0.001) |  | 20 |  | 252.92 (<0.001) |
|  |  |  |  |  |  |  |  |  |  |  |  |  |  |  |  |
| Capacity to work from home X Calendar week | 10 |  | 31.44 (<0.001) |  | 21 |  | 103.11 (<0.001) |  | 14 |  | 110.23 (<0.001) |  | 20 |  | 358.00 (<0.001) |
|  |  |  |  |  |  |  |  |  |  |  |  |  |  |  |  |

Note. All models are calculated for each labour market indicator separately. Models are adjusted for proportion of employees without professional qualification, proportion of female employees,
average income, district type, settlement density, average living space, and boarder region, as well as dummies are included for each calendar week (together with interactions between dummies of calendar weeks and the respective labour market indicator).
